# Epidemiology and natural history of POLG disease in Norway: a nationwide cohort study

**DOI:** 10.1002/acn3.52088

**Published:** 2024-06-07

**Authors:** Erle Kristensen, Linda Mathisen, Siren Berland, Claus Klingenberg, Eylert Brodtkorb, Magnhild Rasmussen, Trine Tangeraas, Yngve T. Bliksrud, Shamima Rahman, Laurence Albert Bindoff, Omar Hikmat

**Affiliations:** ^1^ Department of Medical Biochemistry Oslo University Hospital Oslo 0424 Norway; ^2^ Department of Clinical Medicine (K1) University of Bergen Bergen 5020 Norway; ^3^ Department of Medical Genetics Oslo University Hospital Oslo 0424 Norway; ^4^ Department of Medical Genetics Haukeland University Hospital Bergen 5021 Norway; ^5^ Paediatric Research Group, Department of Clinical Medicine UiT The Arctic University of Norway Tromsø 9019 Norway; ^6^ Department of Paediatrics University Hospital of North Norway Tromsø 9019 Norway; ^7^ Department of Neurology St. Olavs University Hospital Trondheim 7006 Norway; ^8^ Department of Neuromedicine and Movement Science, Faculty of Medicine Norwegian University of Science and Technology Trondheim 7491 Norway; ^9^ Unit for Congenital and Hereditary Neuromuscular Conditions (EMAN), Department of Neurology Oslo University Hospital Oslo 0424 Norway; ^10^ Department of Clinical Neurosciences for Children Oslo University Hospital Oslo 0424 Norway; ^11^ Department of Paediatric and Adolescent Medicine Oslo University Hospital Oslo 0424 Norway; ^12^ European Reference Network for Hereditary Metabolic Disorders; ^13^ Metabolic Unit Great Ormond Street Hospital London UK; ^14^ Mitochondrial Research Group, Genetics and Genomic Medicine Department UCL Great Ormond Street Institute of Child Health London UK; ^15^ Department of Neurology Haukeland University Hospital Bergen 5021 Norway; ^16^ Department of Paediatrics and Adolescent Medicine Haukeland University Hospital Bergen 5021 Norway

## Abstract

**Objective:**

To investigate the prevalence and natural history of POLG disease in the Norwegian population.

**Methods:**

A national, population‐based, retrospective study using demographic, clinical, and genetic data of patients with genetically confirmed POLG disease. The patients were diagnosed between 2002 and 2022, and were included into the Norwegian POLG Patient Registry. Patients were stratified according to age at disease onset (early <12 years, juvenile to adult 12–40 years, late ≥40 years) and resident region.

**Results:**

Ninety‐one patients were included. The point prevalence of POLG disease was 1:149,253. Birth prevalence was 1:48,780. Median age at clinical onset was 16 years (range: 2 months to 70 years). Onset occurred early in 35% (32 out of 91), juvenile‐adult in 55% (50 out of 91) and late in 10% (9 out of 91). A distinct seasonal pattern in disease onset was observed, with 57% (52 out of 91) presenting between May and August. Forty‐five patients (49%) had acute exacerbations that required intensive care, and this affected 72% of those in the early‐onset group. The mortality rate was 54% (49 out of 91), with a median time from disease onset to death of 3 years (range: 1 month to 36 years).

**Interpretation:**

We provide the point prevalence and birth prevalence of POLG disease in the first nationwide study in which epidemiological and clinical data were integrated. Seasonal variations in clinical onset may offer valuable insights into disease mechanisms and modifying factors. The findings from this study are crucial for quantifying the disease burden, and contribute to evidence‐based healthcare planning.

## Introduction

Reliable epidemiological and longitudinal clinical data are essential to understand the socio‐economic impact of a disease and to allocate resources effectively. Epidemiological research on rare disorders poses challenges due to limited data availability, necessitating structured databases. POLG disease is considered to be among the most common primary mitochondrial disorders.^1^, ^2^ However, previous epidemiological studies have almost exclusively investigated specific phenotypes or *POLG* variants or certain age groups.^3^, ^4^, ^5^, ^6^, ^7^, ^8^, ^9^ Extensive national population‐based studies are currently lacking. A previous study conducted on adult patients (> 16 years) in North East England estimated the point prevalence of autosomal‐recessive POLG disease to be 1:333,333.^10^ A study in the Norwegian population found a combined carrier frequency of 1:50 for the *POLG* variants p.Trp748Ser (c.2243G>C) and p.Ala467Thr (c. 1399G>A).^11^ In Finland, the carrier frequency of p.Trp748Ser has been estimated to be 1:125.^6^ By using data from a large in‐house exome database, a recent German study found an allele frequency of pathogenic POLG variants of 0.0074.^12^

The nuclear gene *POLG* encodes the catalytic subunit of DNA polymerase gamma, the enzyme responsible for replication and repair of the mitochondrial DNA (mtDNA). Pathogenic *POLG* variants cause impaired replication and maintenance of the mtDNA and lead to mtDNA point mutations, deletions or depletion, hampering the mitochondrial structure and function. Since the initial discovery of disease‐causing variants in the *POLG* gene in 2001,^13^ it has become well‐established that POLG disease can lead to diverging clinical presentations and multi‐organ involvement.^14^ The mode of inheritance can be both autosomal‐recessive and ‐dominant (AD). AD POLG disease is typically associated with late onset and a mild phenotype. The age of disease onset may serve as an indicator for predicting the key symptoms and the prognosis. Early‐onset patients typically develop a devastating epileptic encephalopathy, sometimes associated with fatal hepatopathy, while myopathy and peripheral neuropathy characterize late‐onset disease. The earlier the onset, the more severe the phenotype.^15^ A considerable proportion of the patients develops intractable epilepsy and status epilepticus, necessitating prolonged admission to intensive care units. Current treatment options are merely symptomatic, and mortality is high.^15^, ^16^, ^17^ Consequently, POLG disease exerts a major burden on affected individuals and families, and represents a substantial healthcare and socio‐economic challenge.

Studying the natural history of the disease will allow characterization of the inherent variability in symptom presentation. This is vital for distinguishing between natural fluctuations of symptoms and treatment effects when evaluating potential future therapies. Our study aimed to assess the national and regional epidemiological characteristics of POLG disease within the Norwegian population. By doing so, we seek to enhance the allocation of healthcare resources, taking into account the distribution and prevalence of this rare disorder. Furthermore, we aimed to delineate the natural history of POLG disease by using demographic, clinical and genetic data derived from a large national cohort, to enhance the awareness of the variable phenotypes of the disorder and to identify potential targets for preventive interventions and future research.

## Methods

### Study design and population

We conducted a retrospective, multicentre cohort study using longitudinal data from the Norwegian POLG Registry. Patients were ascertained from participating centres, as well as the genetic laboratories serving all regions of Norway (Haukeland University Hospital, Oslo University Hospital, St. Olav Hospital and University Hospital of North Norway). The diagnosis was established by genetic laboratories affiliated with the Regional Health Authorities that are the only laboratories performing these investigations. Patients were considered eligible if they were residents of Norway and had confirmed pathogenic *POLG* variants. Deceased patients were included if they had a clinical course compatible with POLG disease and a sibling with confirmed pathogenic *POLG* variants.

The date of disease onset was defined as when symptoms caused by POLG disease led to healthcare contact. Observation time was defined as the time from disease onset to last follow‐up or death. Patients were categorized, as previously described, into early‐onset POLG disease, defined as onset of symptoms before the age of 12 years; juvenile/adult‐onset with onset of symptoms between 12 and 40 years; and late‐onset with symptoms starting after the age of 40 years.^15^

Norway is a large country with a relatively small population that is unequally distributed. Hospitals, serving both the paediatric and adult populations are controlled by four Regional Health Authorities (North, Central, South East and West). South East Norway is the most densely populated part, followed by West Norway. The population sizes of Central and North Norway are smaller, yet comparable, although North Norway is the least densely populated area. Norway has a universal centrally funded healthcare system. Patients were additionally stratified according to the affiliated healthcare region: West Norway, North Norway, South East Norway and Central Norway.

### Data collection

The responsible investigator(s) in each centre completed an electronic case report form including demographic, clinical and genetic data. Inclusion of patients and data entry started in September 2015 and was completed in June 2022. Current survival status of each patient was updated at the time of last data entry.

### Statistical analysis

Data analyses were performed using SPSS (Statistical Product and Service Solutions) version 27.0/29.0. Descriptive measures are reported as numbers (per cent), median (range) and mean (standard deviation and 95% confidence interval for the mean). Survival outcomes were estimated using Kaplan–Meier analyses. Differences in crude survival between the groups were evaluated using the log‐rank test. End point was defined as the time from disease onset to either the date of death or the date of the last follow‐up. Statistical tests were two‐sided, with a significance level determined at less than 0.05.

Birth prevalence was defined as the number of patients with POLG disease born in a specific area during a specified period divided by the total number of live births in the same area and period.^18^ A previous study calculated the birth prevalence for 20 inherited metabolic diseases included into the expanded Norwegian newborn screening program from March 2012, using clinically presenting cases born from March 2002 to February 2012.^19^ To enable comparison with POLG disease, we selected the decade 2002–2011 for birth prevalence calculations. Point prevalence was calculated using the number of alive patients with symptomatic POLG disease at census time (01.01.2016), divided by the total Norwegian or regional population at census time.^18^ Ninety‐five percent confidence intervals (CIs) for prevalence estimates were calculated using the Wilson score interval.

## Results

### Demography

**Figure 1:**
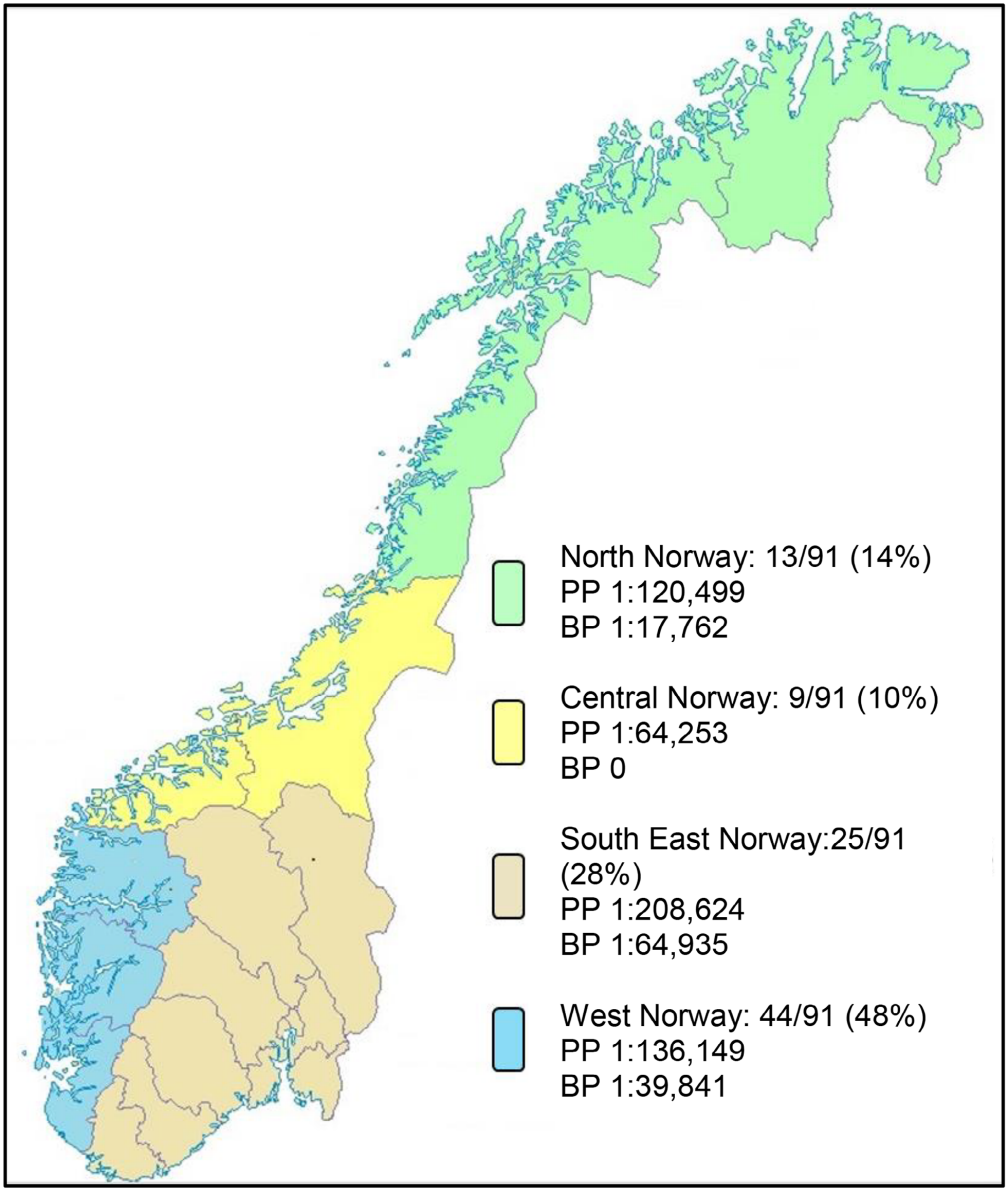
Number of resident patients in the four regions of Norway, point prevalence (PP) at census time 01.01.2016 and birth prevalence (BP) for the decade 2002–2011. Patients with POLG disease/population number at census time and number of patients with POLG disease born/total number of live births 2002–2011^18^: North 4/481,994; 3/53,268. Central 7/449,769; 0/50,812. South East 14/2,920,730; 5/323,248. West 10/1,361,492; 4/159,241. Ninety‐five per cent confidence intervals: North PP (1:309,598–1:46,860), BP (1:52,219–1:6,039). Central PP (1:132,626–1:31,124), BP (0–1:13,228). South East PP (1:349,650–1:124,224), BP (1:151,286–1:27,617). West PP (1:250,627–1:73,964), BP (1:102,354–1:15,482). Map basis: Kartverket (Creative Commons Attribution ShareAlike 3.0).

Ninety‐two patients diagnosed with POLG disease between 2002 and 2022 were recruited to the Norwegian POLG registry. One patient was lost to follow‐up, meaning 50 (55%) males and 41 (45%) females were available for analysis. All were White, except one patient with both Black and White heritage. Nearly half the patients (44 out of 91, 48%) were residents of West Norway, 28% (25 out of 91) lived in South East Norway, 14%(13 out of 91) lived in North Norway and 10% (9 out of 91) in Central Norway (Fig. 1).

### Prevalence estimates

At census time, 35 patients were alive within the total Norwegian population of 5,213,985 individuals, giving a point prevalence of 1:149,253 [95% CI: (1:207,039–1:107,066)]. Within a population of 1,127,402 children (0–17 years), 6 patients were alive, yielding a point prevalence of 1:187,900 [95% CI: (1:409,836–1:86,133)]. Twenty‐nine adult patients (age 18–81 years) were alive within a total population of 3,911,044, giving a point prevalence of 1:134,864 [95% CI: (1:193,798–1:93,897)]. The regional point prevalences are outlined in Figure 1.

Twelve patients were born during the decade 2002–2011 from a total of 586,569 live births, giving a birth prevalence of 1:48,780 [95% CI: (1:85,470–1:27,964)]. This suggests that, on average approximately one person in Norway is born each year who will develop POLG disease during their lifetime. Due to evolving diagnostic possibilities over this cohort's timeline and as we know that there are patients from recent birth cohorts yet to manifest their POLG disease, we included a table displaying decadal birth prevalence and distribution of age of onset within each decade (Table S1).

The birth prevalences of POLG disease and 20 other inherited metabolic diseases are presented in Table 1.

**Table 1:** Birth prevalence for POLG disease patients born in 2002–2011 and patients born in March 2002 to February 2012 clinically diagnosed with inherited metabolic diseases included in the Norwegian newborn screening program from March 2012.

	Birth prevalence
POLG disease	1:48,780
Medium chain acyl‐CoA dehydrogenase deficiency	1:74,573
Tyrosinaemia type 1	1:74,573
Glutaric aciduria type 1	1:99,431
Maple syrup urine disease	1:119,318
Methylmalonic aciduria	1:119,318
Isovaleric aciduria	1:149,147
Long‐chain 3‐hydroxyacyl‐CoA dehydrogenase deficiency	1:149,147
Trifunctional protein deficiency	1:198,863
Carnitine‐acylcarnitine translocase deficiency	1:298,295
Glutaric aciduria type 2	1:298,295
Holocarboxylase synthetase deficiency	1:298,295
Beta‐ketothiolase deficiency	1:596,591
Cystathionine β‐synthase deficiency	1:596,591
Very long‐chain acyl‐CoA dehydrogenase deficiency	1:596,591
Biotinidase deficiency	0
Carnitine palmitoyl‐transferase 1A deficiency	0
Carnitine palmitoyl‐transferase 2 deficiency	0
Carnitine transporter deficiency	0
3‐hydroxy 3‐methylglutaryl‐CoA lyase deficiency	0
Propionic aciduria	0

Table adapted from Tangeraas et al.^19^

### Clinical features

#### Onset of POLG disease

Age of onset ranged from 2 months to 70 years, with a median of 16 years. Mean, standard deviation and 95% confidence intervals for the mean are presented in Table S2. Onset occurred early in 32 out of 91 (35%), juvenile/adult in 50 out of 91 (55%), while 9 out of 91 (10%) presented late. Median age at onset within each age group was 2 years (2 months to 11 years), 19 years (12–39 years) and 53 years (40–70 years), respectively. A higher proportion of the patients in North and West Norway had early‐onset disease compared to Central and South East Norway, while the majority in Central Norway had juvenile/adult onset (Table 2). Onset appeared spontaneously in 71 out of 91 (78%). In 18 out of 91 (20%), onset appeared to be triggered by infection, and of these, 11 had early‐onset disease. Twenty‐nine patients in the juvenile/adult‐onset group were female, and in 9 of these (31%), onset occurred during pregnancy: eight during the second trimester and the last in the third trimester.

**Table 2:** Patient characteristics related to the resident region.

	Total	North	Central	South East	West
Early‐onset	32/91 (35%) (23 M, 9 F)	6/13 (46%)	2/9 (22%)	7/25 (28%)	17/44 (39%)
Juvenile/adult‐onset	50/91 (55%) (21 M, 29 F)	6/13 (46%)	6/9 (67%)	14/25 (56%)	24/44 (55%)
Late‐onset	9/91 (10%) (6 M, 3 F)	1/13 (8%)	1/9 (11%)	4/25 (16%)	3/44 (7%)
Mode of inheritance	H: 48/91 (53%) CH: 39/91 (43%) AD: 4/91 (4%)	H: 7/13 (54%) CH: 6/13 (46%) AD: 0	H: 7/9 (78%) CH: 0 AD: 2/9 (22%)	H: 13/25 (52%) CH: 10/25 (40%) AD: 2/25 (8%)	H: 21/44 (48%) CH: 23/44 (52%) AD: 0
Time from onset to death	3 years (1 month–49 years)	3 years (3 months–20 years)	26 years (3–48 years)	4 months (1 month–27 years)	7 years (1 month–49 years)
Age at death	20 years (8 months–90 years)	18 years (1–61 years)	40 years (21–57 years)	7 years (8 months–78 years)	23 years (8 months–90 years)
Seizures	65/91 (71%)	11/13 (85%)	6/9 (67%)	11/25 (44%)	37/44 (84%)
Tonic–clonic	61/90 (68%)	11/13 (85%)	6/9 (67%)	7/24 (29%)	37/44 (77%)
Myoclonic	44/90 (49%)	7/12 (58%)	4/9 (44%)	6/25 (24%)	27/44 (61%)
Status epilepticus	53/91 (58%)	9/13 (69%)	4/9 (44%)	7/25 (28%)	33/44 (75%)
Focal	62/91 (68%)	10/13 (77%)	6/9 (67%)	11/25 (44%)	35/44 (80%)
EPC	29/89 (33%)	6/12 (50%)	2/9 (22%)	4/25 (16%)	17/43 (40%)
Ataxia	70/91 (77%)	8/13 (62%)	9/9 (100%)	15/25 (60%)	38/44 (86%)
Peripheral neuropathy	56/91 (62%)	8/13 (62%)	8/9 (89%)	12/25 (48%)	28/44 (64%)
Stroke‐like episodes	37/91 (41%)	4/13 (31%)	5/9 (56%)	4/25 (16%)	24/44 (55%)
Migraine	42/91 (46%)	5/13 (39%)	4/9 (44%)	8/25 (32%)	25/44 (57%)
Hypotonia	24/91 (26%)	6/13 (46%)	1/9 (11%)	7/25 (28%)	10/44 (23%)
Weakness	66/91 (73%)	12/13 (92%)	7/9 (78%)	15/25 (60%)	32/44 (73%)
Sensorineuronal hearing loss	10/91 (11%)	1/13 (8%)	1/9 (11%)	3/25 (12%)	5/44 (11%)
Global developmental delay	14/76 (18%)	1/11 (9%)	1/3 (33%)	4/24 (17%)	8/38 (21%)
Ptosis	34/90 (38%)	4/13 (31%)	4/9 (44%)	9/25 (36%)	17/43 (40%)
PEO	43/91 (47%)	6/13 (46%)	8/9 (89%)	12/25 (48%)	17/44 (39%)
Nystagmus	39/91 (43%)	4/13 (31%)	6/9 (67%)	4/25 (16%)	25/44 (57%)
Visual impairment	30/91 (33%)	2/13 (15%)	4/9 (44%)	4/25 (16%)	20/44 (45%)
Anaemia	53/91 (58%)	9/13 (69%)	4/9 (44%)	7/25 (28%)	33/44 (75%)
Feeding difficulties	39/91 (43%)	9/13 (69%)	3/9 (33%)	6/25 (24%)	21/44 (48%)
Hepatic disorder	46/91 (51%)	8/13 (62%)	2/9 (22%)	10/25 (40%)	26/44 (59%)

Numbers represent proportion (per cent) or median (minimum to maximum). H, homozygous; CH, compound heterozygous; AD, autosomal dominant; PEO, progressive external ophthalmoplegia; EPC, epilepsia partialis continua; M, male; F, female.

**Figure 2:**
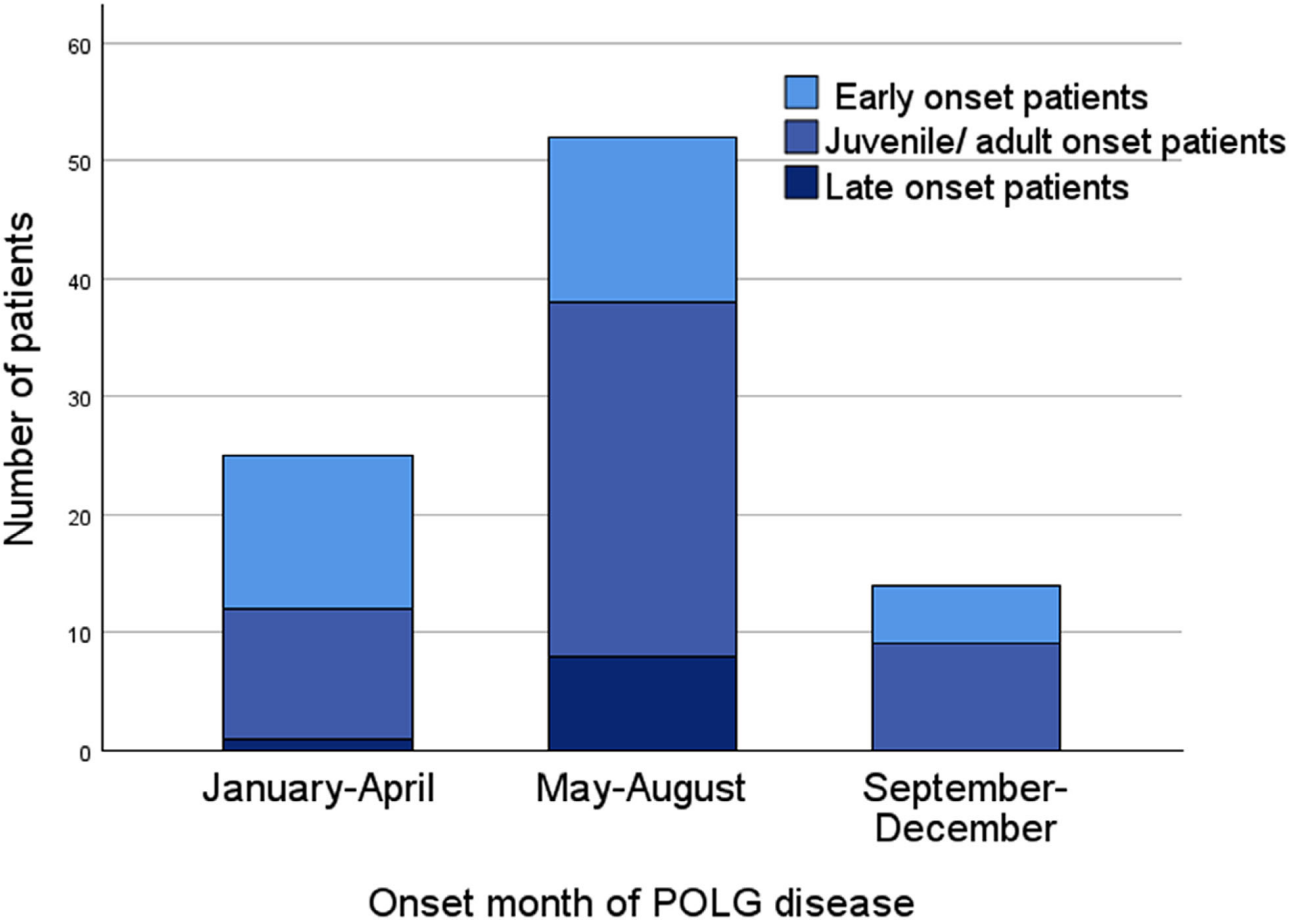
Seasonal variation in clinical onset of POLG disease, with a majority of cases (52 out of 91, 57%) initially presenting between May and August. Stacks represent the age at onset group.

Fifty‐seven per cent (52 out of 91) of the patients had onset of their disease between the months of May and August (Fig. 2). When we broke this down by category, we found that 89% (8 out of 9) late‐onset patients presented during the four summer months as did 60% of the juvenile/adult cases (30 out of 50). The remainder were evenly distributed throughout the year. A different trend emerged in the early‐onset group; similar numbers of cases presented from January to April (13out of 32, 41%) and May to August (14 out of 32, 44%). The impact of seasonal variation showed no discernible regional disparities.

#### Diagnosis

The date of the genetic diagnosis was known in 76 (84%) patients. In two patients with affected siblings, POLG disease was ascertained presymptomatically, respectively 1 week and 8 months before disease onset. In the remaining, the median time from disease onset to diagnosis was 9 years (4 days to 49 years). In patients with onset before 2005, the diagnosis was ascertained with a median time of 18 years (5 months to 49 years). Patients with onset from 2005 to 2022 received a genetic diagnosis at a median of 4 months (4 days to 9 years). Among the patients who presented from 2015 to 2022, the diagnosis was ascertained at a median time of 2 months (4 days to 6 years).

#### Major clinical features

**Figure 3:**
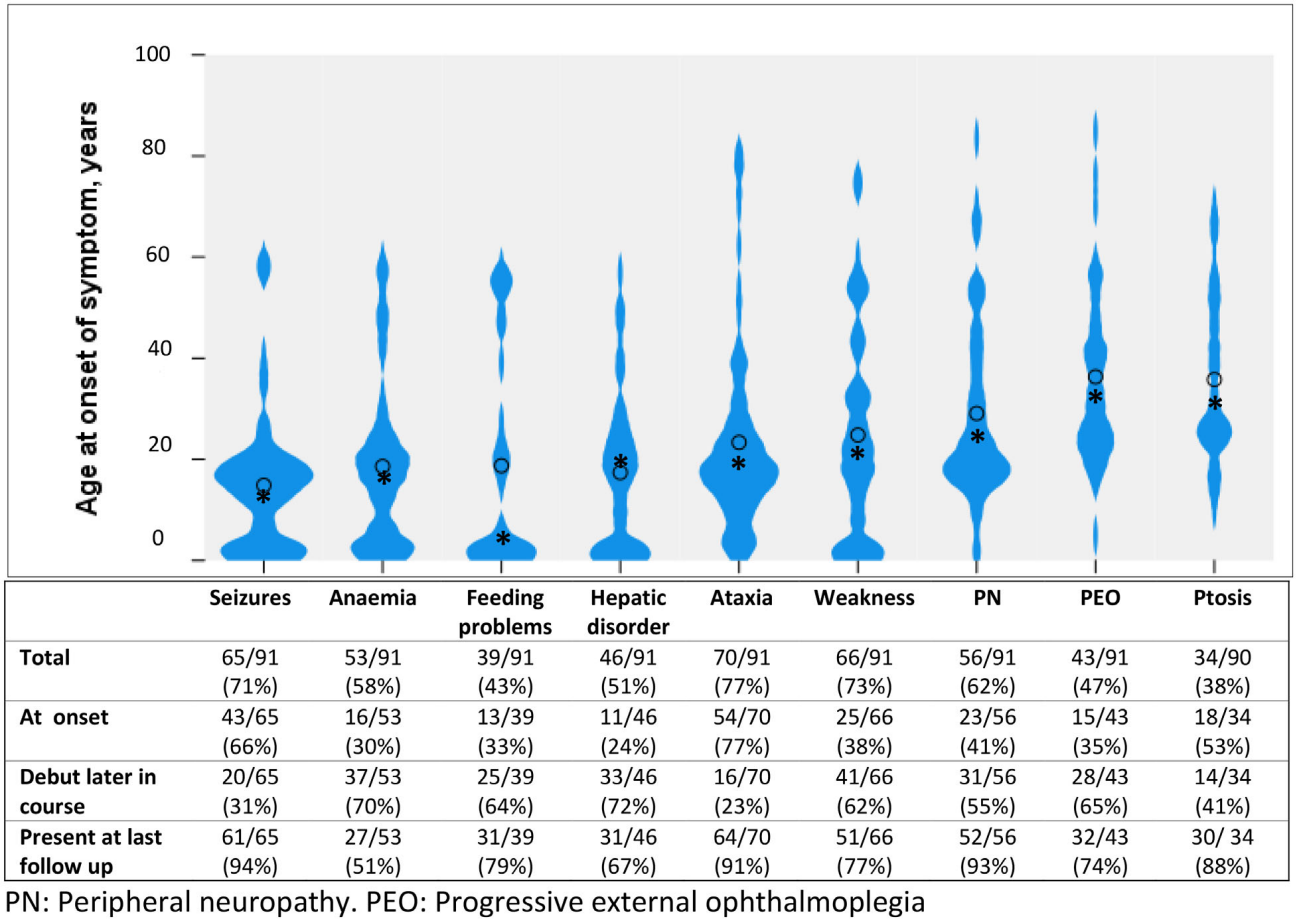
Violin plots representing age at initial presentation of some of the major clinical features. Dot: mean age at onset of symptom, asterisk: median age at onset of symptom. The table displays the proportion (per cent) of patients who exhibited each symptom at the onset of POLG disease or developed each symptom later in the disease course and the number of patients in whom the symptom was present at the last follow‐up. The onset of the different clinical features could appear across the entire lifespan, although most showed a noticeable trend. While ataxia and seizures were common at the presentation of POLG disease, weakness, hepatic disorder and anaemia typically appeared later during the disease course. In general, most of the symptoms outlined persisted at the last follow‐up.

A broad spectrum of clinical features was found, with ataxia, weakness and seizures being the most common. While anaemia was seen in more than half the patients (53 out of 91, 58%), leukopaenia was reported only in one, and thrombocytopaenia in none. The most frequent clinical features seen differed according to age of onset. In the early‐onset group, seizures (31 out of 32, 97%), anaemia (25 out of 32, 78%) and feeding difficulties (23 out of 32, 72%) were the dominant symptoms. In the juvenile/adult‐onset group, ataxia (45 out of 50, 90%), weakness (38 out of 50, 76%) and peripheral neuropathy (37 out of 50, 74%) were most prevalent, and in the late‐onset group, progressive external ophthalmoplegia (9 out of 9, 100%), ataxia (8 out of 9, 89%) and ptosis (8 out of 9, 89%). Clinical characterization of the patients within the different regions is presented in Table 2, and acquisition of clinical features at debut and follow‐up in Figure 3.

Fifty‐two patients (57%) experienced acute exacerbations that required hospitalization, of these 45 were admitted to the intensive care unit. Among the early‐onset patients, 23 out of 32 (72%) received intensive care, while this applied to 21 out of 50 (42%) juvenile/ adult‐onset patients and one late‐onset patient. Seizures were the most common type of exacerbation.

Clinical features frequently associated with other mitochondrial disorders, such as cataracts (5 out of 91, 5%), pigmentary retinopathy (1 out of 91, 1%) and Type II diabetes mellitus (1 out of 91, 1%), were uncommon or absent (cardiomyopathy 0 out of 91).

### Genetic results

Among the 15 *POLG* variants detected in this cohort, the founder variants NM_002693.3(POLG):c.2243G>C (p.Trp748Ser) and NM_002693.3(POLG):c.1399G>A (p.Ala467Thr) contributed 103 out of 174 and 44 out of 174 of the recessive alleles, respectively (Figure S1). While the majority of these were homozygous (48 out of 91, 53%), 39 out of 91 (43%) were compound heterozygous. Only 4 out of 91 (4%) patients had an AD inheritance, all carrying the p.Tyr955Cys (c. 2864A>G) variant. AD disease was seen in 3 out of 50 (6%) in the juvenile/ adult‐onset group and in 1 out of 9 (11%) in the late‐onset group. The regional distribution of mode of inheritance is presented in Table 2, whereas the regional distribution of *POLG* variants is depicted in Figure S1 and the regional distribution of *POLG* genotypes is outlined in Table S3.

### Survival

At the time of data entry, 49 patients (54%) were deceased. Median age at death was 20 years (8 months to 90 years), and median time from disease onset to death was 3 years. Of those with early‐onset disease, 22 out of 32 (69%) were dead: median age at death was 2 years (8 months to 57 years), and the median time from onset to death was 8 months (1 month to 49 years). In 24 out of 50 (48%) patients with juvenile/adult‐onset, death occurred at a median age of 24 years (12–57 years) with a median time of 7 years (4 months to 36 years) from onset to death. In the late‐onset group 3 out of 9 (33%) were deceased, with a median age of 79 years (61–90 years), and a median time from onset to death of 26 years (21–28 years).

When examining regional disparities, we observed a higher mortality rate among patients in North (10 out of 13, 77%) and West Norway (27 out of 44, 61%) compared to Central and South East Norway (Table 2). Survival analysis revealed significantly reduced survival in North and West compared to Central Norway (*P* = 0.005 and *P* = 0.033, respectively) (Figure S2).

Sepsis was the major cause of death (15 out of 49, 31%), followed by liver failure (12 out of 49, 25%) and status epilepticus (11 out of 49, 22%). Among the 15 patients who died of sepsis, 9 were registered with hepatic disorder and 13 with seizures at the time of death. Four patients died from multiple organ failure, all in the early‐onset group and with seizures at the time of death. The cause of death remained unknown in 12% (6/49).

## Discussion

### Epidemiology

This study is the first national, population‐based cohort study of POLG disease and it provides novel and essential insights into its prevalence and socio‐economic impact. Utilizing data from our national POLG disease registry, we estimated a point prevalence of 1:149,253 and a birth prevalence of 1:48,780. Norway boasts a universal, publicly funded healthcare system that facilitates genetic investigations and care for all residents. This contributes to a comprehensive overview of patients with POLG disease in Norway. Thus, we argue that we have a nearly complete inclusion of diagnosed patients.

Patients with mitochondrial disorders often require multidisciplinary healthcare services and pose a substantial economic cost on society.^20^, ^21^ Acute and severe exacerbations may necessitate prolonged hospital admissions. Adults with mitochondrial disorders display an average monthly healthcare cost seven times that of the general adult population, which is comparable to the costs for multiple sclerosis and amyotrophic lateral sclerosis.^21^ Considering that POLG disease presents with greater severity in early‐onset patients, it is likely that paediatric patients need more frequent and extended hospitalizations necessitating intensive care, resulting in a higher demand for healthcare resources when compared to adults.^15^ Indeed, 72% of the early‐onset patients experienced acute exacerbations that required intensive care. The aforementioned study revealed a healthcare cost for children with mitochondrial disease 23 times higher than the paediatric population's average.^21^ Elucidating the natural history of the disorder in conjunction with prevalence estimates is essential to form a comprehensive understanding of the disease's overall burden. Hence, point prevalence is an indispensable measure to estimate healthcare costs and resources.

Compared to a prior study that identified a point prevalence of 1:333,333 in adult patients with POLG disease,^10^ our current study revealed a higher point prevalence of 1:134,864 among adults. Including individuals of all ages into our registry allowed us to calculate a point prevalence of 1:187,900 for paediatric patients. The slight difference in point prevalence between the adult and paediatric patient population may in part reflect the higher mortality in children.

Central Norway displayed a point prevalence over three times higher than South East Norway and more than twice as high as West Norway. This could be a manifestation of the significantly better survival in this region than in West and North. This discrepancy may reflect the finding that all patients in Central Norway either had AD or homozygous p.Trp748Ser recessive disease (Table S3) that have been associated with later onset and milder form of disease.^15^, ^16^, ^22^, ^23^, ^24^ AD inheritance was not seen in North and West Norway. Here, the proportion of patients with early‐onset disease, which is associated with poor outcomes was higher. Moreover, clinical features associated with worse survival, such as hepatic disorder, anaemia and epilepsy,^15^, ^25^ were more prevalent in these regions. These prognostically unfavourable features were also reflected in the reduced survival in North and West compared to Central Norway.

Our study showed a birth prevalence of POLG disease of 1: 48,780. Birth prevalence not only provides a standardized measure widely used in estimating the occurrence of genetic disorders, but also, by applying the Hardy–Weinberg principle, allows comparison with allele and carrier frequency calculations. We found that POLG disease had a birth prevalence 1.5 times higher than clinically presenting MCADD and tyrosinaemia type 1 (Table 1). This could imply that POLG disease is among the most common inherited metabolic diseases in Norway, and emphasizes the significance of clinical awareness.

We found regional discrepancies with regard to birth prevalence. In North Norway, the birth prevalence was nearly four times higher than in South East and more than twice that in West. Even if this to some degree may reflect random variations, it indicates differences within Norway that could be caused by regional genetic distinctions. No patients with POLG disease were born in Central Norway during 2002–2011, the period used for computation of birth prevalence. All patients registered in Central Norway were born before 2002 and displayed a generally mild clinical phenotype. This may reflect the type of POLG disease prevalent in Central Norway, namely AD or homozygous recessive disease, and that patients from the birth cohort 2002–2011 are yet to manifest.

Prevalence studies based on calculating carrier or allele frequencies from small sample sizes face the challenge of random accumulations of disease alleles, which can lead to unreliable results. Applying the Hardy–Weinberg principle to a previous Norwegian study, which involved carrier frequency data of two *POLG* founder variants (p.Trp748Ser and p.Ala467Thr) obtained from 98 healthy control individuals, yields a birth prevalence of POLG disease of 1:10,000.^11^ This predicts approximately five children with POLG disease attributed solely to these genetic variants would be born in Norway each year. This estimate is however compromised by the small sample size. Larger exome or genome databases may provide more representative carrier frequencies, making random accumulations of disease alleles less likely. The aforementioned study by Tan et al.^12^ calculated a lifetime risk of POLG disease of 3.7/100,000. The discrepancy to our estimated birth prevalence of 1:48,780 might result from the loss of fetuses carrying biallelic POLG variants or patients dying from acute decompensations before POLG disease was even suspected, or it may be ascribed to patients who remain undiagnosed due to inconspicuous symptoms.

### Natural history

The median duration from disease onset to the final follow‐up or date of death was 9 years (3 weeks to 49 years), affording this study a cumulative observational period of 1005 person‐years. This extensive data set serves as a robust basis for elucidating the natural history of POLG disease. Our findings confirm that POLG disease is a heterogeneous disease that can present at any age and usually involves several organ systems, with any type of the clinical features presenting throughout the lifespan. This underlines the conclusion that the clinical phenotype in POLG disease forms a continuous spectrum rather than separate entities, and that there is a certain chronology in the appearance of symptoms (Fig. 3).

The median time from disease onset to genetic diagnosis was 9 years. The time required to confirm diagnosis decreased dramatically to a median of 4 months for patients with clinical onset after 2005, and to 2 months for those who presented after 2015. We believe that the promptness of diagnostic ascertainment in patients with POLG disease can be attributed to improved awareness, as well as the inclusion of the *POLG* gene in a wide range of gene panels, and the increasing availability and affordability of genetic analyses in recent years.

Given the propensity of POLG disease to present acutely and its severity, we sought for trigger factors precipitating onset. Among female patients with juvenile/adult onset of POLG disease, 31% presented during pregnancy. Puberty and pregnancy have previously been shown to exert a negative impact on female patients, triggering both disease onset and deterioration mainly manifested by seizure exacerbation.^26^ Both increased energy demands during pregnancy and hormonal influences on neuronal excitability have been proposed as contributing factors. In the early‐onset patients, disease onset was more strongly associated with infection than those with onset after 12 years. Infection is a state of elevated energy demands, imposing increased pressure on the oxidative phosphorylation system as well as induction of the immune system and has previously been reported as both an initiating factor and a catalyst of disease exacerbations in mitochondrial disorders.^27^, ^28^ In POLG disease, oligoclonal bands as well as increased cytokines in the cerebrospinal fluid have been reported, indicating an immunologic component.^29^, ^30^ Our data did not provide details on what type of infection triggered the onset of disease, but in previous reports, viral infections, as well as Borrelia infection, have been associated with the onset of POLG disease.^31^, ^32^ Surprisingly, irrespective of infectious disease, we observed a noticeable seasonal pattern in disease onset, with a pronounced peak between May and August (Fig. 2). This finding is striking given that the onset of monogenic disorders does not typically show an overt association with seasonal changes. To our knowledge, such a phenomenon has not been described in POLG disease nor any other mitochondrial disorder. Seasonal variation is well known in several neurological disorders, relapse in multiple sclerosis being perhaps the most noteworthy example.^33^, ^34^ Metabolite investigations in patients with multiple sclerosis have further supported seasonal fluctuations affecting both carbohydrate, lipid and amino acid metabolism.^35^ Seasonal fluctuations are primarily associated with environmental factors and other variables subject to annual changes, like infections and diet. Nevertheless, annual variations in endogenous factors, including endocrine influences and temperature might also exert an impact. The observed seasonal differences in onset of the disease in our study may imply the involvement of environmental factors or endogenous seasonal variations in metabolic processes. This important discovery warrants further investigations, as it has the potential to elucidate underlying pathogenic mechanisms of POLG disease. Moreover, it may offer valuable insights that may credit the prevention of specific symptom onset and progression.

### Strengths and limitations

This study provides extensive data from a large national cohort of patients with POLG disease, allowing us to estimate its prevalence. Nonetheless, clinical prevalence estimates are limited by potential ascertainment bias, such as the underreporting of milder or atypical cases, the exclusion of patients who succumbed to the disease prior to identification of its genetic cause and the unavailability of patients who have not yet presented clinically. Before the routine use of gene panels, regional differences in access to genetic testing may have led to missed cases. It is tentative to suggest that there are regional differences in prevalence within the Norwegian population, but in order to draw conclusions on this, larger cohorts must be examined. The limited number of patients in the population of Central Norway and those with late‐onset disease promote a degree of uncertainty, and caution is needed when interpreting these results. Migration, both from abroad and within Norway, may influence birth prevalence. Our analyses were based on residents' regional affiliation, not considering migration patterns.

### Concluding remarks

The point prevalence and birth prevalence of POLG disease found in this study suggest that POLG disease may be among the most prevalent inherited metabolic diseases in Norway. Our findings underscore this disorder's economic impact on society and the personal burden it imposes on the patients and their families. To gain deeper understanding of POLG disease, it is crucial to conduct further investigations into both environmental and endogenous factors that trigger disease onset and exacerbations, including metabolite profiling during different seasons. Establishing patient registries for rare diseases is essential for monitoring the diagnosed individuals count and to acquire comprehensive longitudinal data, in order to facilitate targeted healthcare planning and to incite research on treatment.

## Author contributions

Study design was performed by O.H and E.K. Data analysis, writing of the original draft and visualization was performed by E.K and O.H. Data collection was performed by O.H. and E.K. Data acquisition and critical revision of the manuscript was performed by L.M., S.B., C.K., E.B., M.R., T.T., Y.T.B., S.R. and L.B. Project administration was carried out by O.H. All authors have read and approved the final version of the manuscript.

## Conflict of interest

The authors declare no financial or other conflicts of interest related to this work.

## Supporting information


**Figure S1.** The count of alleles containing the most frequently reported genetic variants, organized by region. The predominant variant observed across all regions was p.Thr748Ser (c.2243G>C), except for Central Norway, where p.Ala467Thr (c.1399G>A) was most prevalent. P.Thr251Ile (c.752C>T) and p.Gly303Arg (c.907G>A) constituted 93 six and seven of the recessive alleles, respectively. Other variants reported: North Norway: p.Arg807Cys (c. 2419C>T) in three alleles, p.Gly268Ala (c. 803G>C) in two alleles and p. Gly1052Asp (c. 3155G>A) in one allele. Central Norway: No other variants reported. South East Norway: p.Arg807Cys (c. 2419C>T), p. Arg574Trp (c. 1720C>T), Phe749Ser (c. 2246T>C) and p. Arg1096Cys (c. 3286C>T), all present in one allele each. West Norway: p. Gly737Arg (c. 2209G>C), p. Gly23Serfs*236 (c. 67_88del22), p. Gln60Ter (c. 178C>T) and p. Gly848Ser (c. c.2542G>A), all present in one allele each.
**Figure S2.** Survival analysis. Kaplan–Meier plot comparing survival in relation to resident region (*P* = 0.05).
**Table S1.** Birth prevalence of POLG disease for decadal birth cohorts.^18^ Ninety‐five percent confidence intervals in brackets. Apart from a birth prevalence of 1:100,049 from 1990 to 1999, birth prevalence varied between 1:35,180 and 1:58,419 in the decades from 1960 to 2019. Twenty‐three of the 32 early‐onset patients (72%) were born during 2000–2019. No late‐onset patients were registered in the birth cohorts after 1970. Seventy percent of the juvenile/adult‐onset patients were born from 1960 to 1989. *Birth numbers are only available from 1922. ** Cumulative birth prevalence for the entire cohort.
**Table S2.** Presentation of median (range), mean, standard deviation and 95% confidence intervals for the variables presented in the article.
**Table S3.** POLG genotypes in each region of Norway.
