# Supplementary material for: Epidemiology and natural history of POLG disease in Norway: a nationwide cohort study
Source: Ann Clin Transl Neurol. 2024 Jun 7;11(7):1819–30. doi: 10.1002/acn3.52088 (PMC11251482; doi:10.1002/acn3.52088)
Supplement: Supplementary file 1 — Figure S1. The count of alleles containing the most frequently reported genetic variants, organized by region. The predominant variant observed across all regions was p.Thr748Ser (c.2243G>C), except for Central Norway, where p.Ala467Thr (c.1399G>A) was most prevalent. P.Thr251Ile (c.752C>T) and p.Gly303Arg (c.907G>A) constituted 93 six and seven of the recessive alleles, respectively. Other variants reported: North Norway: p.Arg807Cys (c. 2419C>T) in three alleles, p.Gly268Ala (c. 803G>C) in two alleles and p. Gly1052Asp (c. 3155G>A) in one allele. Central Norway: No other variants reported. South East Norway: p.Arg807Cys (c. 2419C>T), p. Arg574Trp (c. 1720C>T), Phe749Ser (c. 2246T>C) and p. Arg1096Cys (c. 3286C>T), all present in one allele each. West Norway: p. Gly737Arg (c. 2209G>C), p. Gly23Serfs*236 (c. 67_88del22), p. Gln60Ter (c. 178C>T) and p. Gly848Ser (c. c.2542G>A), all present in one allele each. Figure S2. Survival analysis. Kaplan–Meier plot comparing survival in relation to resident region (P = 0.05). Table S1. Birth prevalence of POLG disease for decadal birth cohorts. 18 Ninety‐five percent confidence intervals in brackets. Apart from a birth prevalence of 1:100,049 from 1990 to 1999, birth prevalence varied between 1:35,180 and 1:58,419 in the decades from 1960 to 2019. Twenty‐three of the 32 early‐onset patients (72%) were born during 2000–2019. No late‐onset patients were registered in the birth cohorts after 1970. Seventy percent of the juvenile/adult‐onset patients were born from 1960 to 1989. *Birth numbers are only available from 1922. ** Cumulative birth prevalence for the entire cohort. Table S2. Presentation of median (range), mean, standard deviation and 95% confidence intervals for the variables presented in the article. Table S3. POLG genotypes in each region of Norway. [file ACN3-11-1819-s001.docx]

**Supplementary Table 1**

| Year of birth | Birth prevalence | Number of patients born | | | |
| --- | --- | --- | --- | --- | --- |
|  |  | **Total** | **Early-onset** | **Juvenile**  **Adult-onset** | **Late-onset** |
| 1922-1929* | 1:454,545 (1:2,487,562, 1:77,580) | 1 | - | - | 1 |
| 1930-1939 | 1:222,222 (1:806,452, 1:60,680) | 2 | - | - | 2 |
| 1940-1949 | 1:99,010 (1:216,450, 1:45,475) | 6 | 2 | 1 | 3 |
| 1950-1959 | 1:89,718 (1:185,185, 1:43,459) | 7 | - | 5 | 2 |
| 1960-1969 | 1:38,280 (1:61,312, 1:23,901) | 17 | 2 | 14 | 1 |
| 1970-1979 | 1:52,662 (1:94,340, 1:29,403) | 11 | 1 | 10 | - |
| 1980-1989 | 1: 35,180 (1:58,038, 1:21,322) | 15 | 4 | 11 | - |
| 1990-1999 | 1:100,049 (1:218,340, 1:45,851) | 6 | - | 6 | - |
| 2000-2009 | 1:36,302 (1:58,962, 1:22,346) | 16 | 13 | 3 | - |
| 2010-2019 | 1: 58,419 (1:107,527, 1:31,736) | 10 | 10 | - | - |
| 1923-2019** | 1:61,350 (1:75,075, 1:49,826) | 91 | 32 | 50 | 9 |

**Supplementary table 2**

| **Variable** | | **Median**  **(Range)** | **Mean** | **SD** | **95% CI** |
| --- | --- | --- | --- | --- | --- |
| Age at onset POLG disease (years) | | 16  (0.2-70) | 18.4 | 16.0 | (15.0, 21.7) |
| Age of onset POLG disease, early onset group (years) | | 2 (0.2-11) | 3.7 | 3.5 | (2.4, 5.0) |
| Age onset POLG disease, juvenile/ adult onset group (years) | | 19 (12-39) | 21.2 | 7.3 | (19.1, 23.3) |
| Age onset POLG disease, late onset group (years) | | 53 (41-70) | 54.8 | 10.0 | (47.1, 62.5) |
| Time from onset to diagnosis (except two patients diagnosed presymptomatically) (years) | All | 9 (4 days-49) | 10.5 | 10.5 | (8.0, 12.9) |
|  | Onset before 2005 | 18 (0.4-49) | 16.6 | 9.6 | (13.6, 19.6) |
|  | Onset 2005-2022 | 0.3 (4 days-9) | 2.0 | 3.1 | (0.8, 3.1) |
|  | Onset 2015-2022 | 0.2 (4 days-6) | 1.2 | 2.1 | (0.02, 2.3) |
| Age at death, all (years) | | 20 (0.7-90) | 24.3 | 22.2 | (17.9, 30.7) |
| Age at death, early onset group (22/32) (years) | | 2 (0.7-57) | 12.3 | 18.3 | (4.2, 20.4) |
| Age at death, juvenile/adult onset (24/50) (years) | | 24 (12-57) | 28.7 | 13.7 | (22.9, 34.5) |
| Age at death late onset (3/9) (years) | | 79 (61-90) | 76.7 | 14.4 | (40.9, 112.5) |
| Age at death, North (years) | | 18 (1-61) | 24.7 | 24.7 | (7.0, 42.3) |
| Age at death, Central (years) | | 40 (21-57) | 39.7 | 25.6 | (-190.6, 270.0) |
| Age at death, South East (years) | | 7 (0.7-78) | 17.5 | 25.3 | (-0.6, 35.7) |
| Age at death, West (years) | | 23 (0.7-90) | 25.5 | 20.4 | (17.5, 33.6) |
| Time from disease onset to death, all (years) | | 3 (0.1-49) | 10.0 | 13.2 | (6.2, 13.8) |
| Time from disease onset to death, early onset group (years) | | 0.7 (0.1-49) | 8.3 | 15.7 | (1.4, 15.3) |
| Time from disease onset to death, juvenile/ adult onset group (years) | | 7 (0.4, 36) | 9.7 | 10.3 | (5.4, 14.1) |
| Time from disease onset to death, late onset group (years) | | 26 (21-28) | 24.7 | 3.7 | (15.6, 33.9) |
| Time from disease onset to death, North (years) | | 3 (0.3-20) | 7.9 | 9.1 | (1.4, 14.3) |
| Time from disease onset to death, Central (years) | | 26 (3-48) | 26.0 | 32.4 | (-265.1, 317.0) |
| Time from disease onset to death, Southeast (years) | | 0.4 (0.1-27) | 5.2 | 9.6 | (-1.7, 12.1) |
| Time from disease onset to death, West (years) | | 7 (0.1-49) | 11.4 | 13.7 | (6.0, 16.8) |

**Supplementary table 3**

| **Genotype** | **North** | **Central** | **South East** | **West** | **Total** |
| --- | --- | --- | --- | --- | --- |
| Homozygous p.Trp748Ser | 2 (15%) | 7 (78%) | 12 (48%) | 17 (39%) | 38 (42%) |
| Homozygous p.Ala467Thr | 4 (31%) | 0 | 1 (4%) | 4 (9%) | 9 (10%) |
| Compound heterozygous p.Trp748Ser, p.Ala467Thr | 1 (8%) | 0 | 2 (8%) | 12 (27%) | 15 (17%) |
| Compound heterozygous p.Ala467Thr, p.Gly303Arg | 1 (8%) | 0 | 0 | 6 (14%) | 7 (8%) |
| Compound heterozygous p.Trp748Ser, p.Thr251Ile | 0 | 0 | 5 (20%) | 0 | 5 (6%) |
| Compound heterozygous, p.Trp748Ser, p.Arg807Cys | 3 (23%) | 0 | 1 (4%) | 0 | 4 (4%) |
| AD disease, heterozygous p.Tyr955Cys | 0 | 2 (22%) | 2 (8%) | 0 | 4 (4%) |
| Compound heterozygous, other variants | 1 (8%) | 0 | 2 (8%) | 5 (11%) | 8 (9%) |
| Homozygous, other variants | 1 (8%) | 0 | 0 | 0 | 1 (1%) |
| **Total** | 13 | 9 | 25 | 44 | 91 |

**Supplementary figure 1**


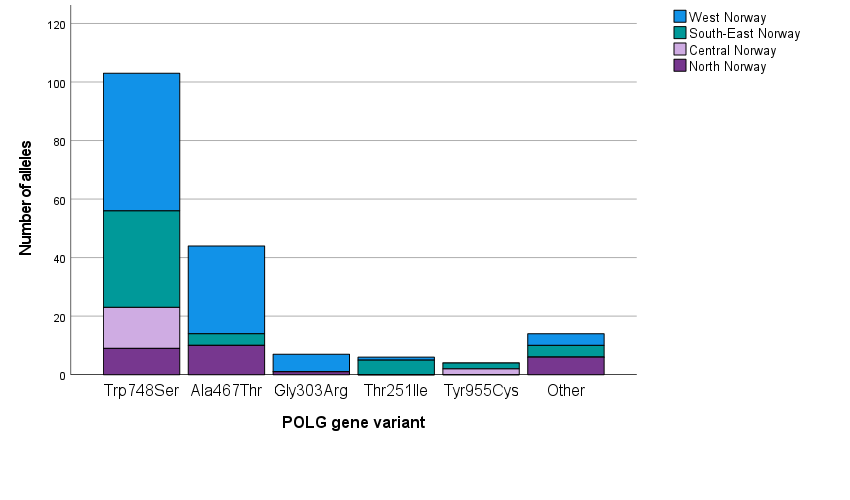


**Supplementary figure 2**


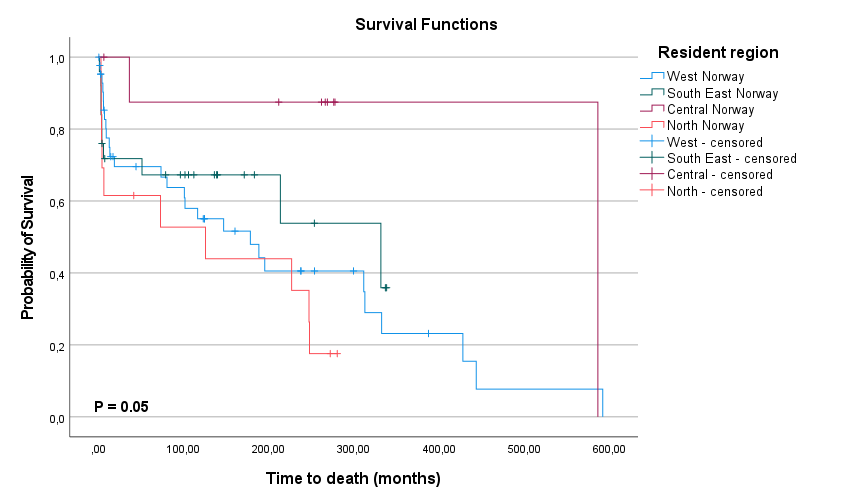


***POLG* gene variant**
